# Supplementary material for: A conserved RNA degradation complex required for spreading and epigenetic inheritance of heterochromatin
Source: eLife. 2020 Jun 3;9:e54341. doi: 10.7554/eLife.54341 (PMC7269676; doi:10.7554/eLife.54341)
Supplement: Supplementary file 1. — This Table outlines the list of fission yeast S. pombe strains that were used in this study and notes the specific figure in the paper in which each strain was used. [file elife-54341-supp1.docx]

**Table S1. List of *S. pombe* strains used in this study.**

| SPY9851 | *h- leu1-32 ade6-M216 ura4Δ::10xtetO-ade6 trp1: nat-clr4p-NLS-TetR-2xflag-clr4ΔCD epe1Δ::kanMX6* | Ref. 1 (SPY5088) | Figure 1  Figure 1Supp1  Figure 2B,D,E  Figure 3A,B,C  Figure 2Supp3  Figure 3Supp2A  Figure 4A,B  Figure 4C,D  Figure 4Supp1 |
| --- | --- | --- | --- |
| SPY9852 | *h+ leu1-32 ade6-M216 ura4Δ::10xtetO-ade6 trp1: nat-clr4p-NLS-TetR-2xflag-clr4ΔCD epe1Δ::kanMX6* | Ref. 1 (SPY5089) | Figure 1  Figure 1Supp1  Figure 3G  Figure 3Supp1A  Figure 3Supp2C |
| SPY9853 | *h- leu1-32 ade6-M216 ura4Δ::10xtetO-ade6 trp1: nat-clr4p-NLS-TetR-2xflag-clr4ΔCD epe1Δ::kanMX6 crb3Δ::crb3-L389P-ura4-hphMX6* | This study | Figure 2Supp3 |
| SPY9854 | *h- leu1-32 ade6-M216 ura4Δ::10xtetO-ade6 trp1: nat-clr4p-NLS-TetR-2xflag-clr4ΔCD epe1Δ::kanMX6 crb3Δ::crb3-D198N isolate 1* | This study | Figure 2B,D,E  Figure 2Supp3 |
| SPY9855 | *h- leu1-32 ade6-M216 ura4Δ::10xtetO-ade6 trp1: nat-clr4p-NLS-TetR-2xflag-clr4ΔCD epe1Δ::kanMX6 crb3Δ::crb3-D198N isolate 2* | This study | Figure 2B,D,E  Figure 2Supp3 |
| SPY9856 | *h- leu1-32 ade6-M216 ura4Δ::10xtetO-ade6 trp1: nat-clr4p-NLS-TetR-2xflag-clr4ΔCD epe1Δ::kanMX6 grc3Δ::grc3-V70M isolate 3* | This study | Figure 2B,D,E  Figure 3A,B,C  Figure 2Supp3 |
| SPY9857 | *h- leu1-32 ade6-M216 ura4Δ::10xtetO-ade6 trp1: nat-clr4p-NLS-TetR-2xflag-clr4ΔCD epe1Δ::kanMX6 grc3Δ::grc3-V70M isolate 1* | This study | Figure 2B,D,E  Figure 2Supp3 |
| SPY9858 | *h- leu1-32 ade6-M216 ura4Δ::10xtetO-ade6 trp1: nat-clr4p-NLS-TetR-2xflag-clr4ΔCD epe1Δ::kanMX6 grc3Δ::grc3-G409E isolate 1* | This study | Figure 2B,D,E  Figure 4C,D  Figure 2Supp3 |
| SPY9859 | *h- leu1-32 ade6-M216 ura4Δ::10xtetO-ade6 trp1: nat-clr4p-NLS-TetR-2xflag-clr4ΔCD epe1Δ::kanMX6 grc3Δ::grc3-G409E isolate 2* | This study | Figure 2B,D,E  Figure 2Supp3 |
| SPY78 | *KGP425 h- leu1-32 ade6-M210 ura4-D18 his3-Dr* | C. Hoffman | Figure 2Supp2 A,B  Figure 3Supp1 C,D,E |
| SPY76 | *fWP44 h+ leu1-32 ade6-M216* | C. Hoffman | Figure 2Supp2 A,B |
| SPY6704 | *h90 ade6? ura4-DS/E CenH(HindIII):ura4* | Ref. 2 | Figure 2Supp2 A |
| SPY9860 | *h90 leu1-32 ade6? ura4Δ:10xtetO-ade6 CenH(HindIII):ura4 crb3Δ::crb3-D198N* | This study | Figure 2Supp2 A |
| SPY9861 | *h+ otr1R(SphI)::ura4 ura4-DS/E leu1-32 ade6-M210* | Ref. 3 | Figure 2Supp2 B |
| SPY9863 | *h+ otr1R(SphI)::ura4 ura4-DS/E leu1-32 ade6-M210 crb3Δ::crb3-D198N isolate 2* | This study | Figure 2Supp2 B |
| SPY9864 | *h+ otr1R(SphI)::ura4 ura4-DS/E leu1-32 ade6-M210 crb3Δ::crb3-D198N isolate 3* | This study | Figure 2Supp2 B |
| SPY9865 | *h+ leu1-32 ade6-M216* *ura4Δ::10xtetO-ade6 trp1: nat-clr4p-NLS-TetR-2xflag-clr4ΔCD epe1Δ::kanMX6 crb3Δ::crb3-WT-TAP isolate 1* | This study | Figure 3A,B,C  Figure 3G  Figure 3Supp1 A,B  Figure 3Supp2C |
| SPY9866 | *h+ leu1-32 ade6-M216* *ura4Δ::10xtetO-ade6 trp1: nat-clr4p-NLS-TetR-2xflag-clr4ΔCD epe1Δ::kanMX6 crb3Δ::crb3-WT-TAP isolate 2* | This study | Figure 3G  Figure 3Supp1A  Figure 3Supp2C |
| SPY9867 | *h+ leu1-32 ade6-M216* *ura4Δ::10xtetO-ade6 trp1: nat-clr4p-NLS-TetR-2xflag-clr4ΔCD epe1Δ::kanMX6 crb3Δ::crb3-WT-TAP grc3Δ::grc3-V70M isolate 2* | This study | Figure 3A,B,C  Figure 3G  Figure 3Supp1B  Figure 3Supp2C |
| SPY9868 | *h+ leu1-32 ade6-M216* *ura4Δ::10xtetO-ade6 trp1: nat-clr4p-NLS-TetR-2xflag-clr4ΔCD epe1Δ::kanMX6 crb3Δ::crb3-WT-TAP grc3Δ::grc3-V70M isolate 1* | This study | Figure 3G  Figure 3Supp2C |
| SPY9869 | *h- leu1-32 ade6-M216* *ura4Δ::10xtetO-ade6 trp1: nat-clr4p-NLS-TetR-2xflag-clr4ΔCD epe1Δ::kanMX6 crb3Δ::crb3-WT-TAP isolate 1* | This study | Figure 3-Supp2 A |
| SPY9870 | *h- leu1-32 ade6-M216* *ura4Δ::10xtetO-ade6 trp1: nat-clr4p-NLS-TetR-2xflag-clr4ΔCD epe1Δ::kanMX6 crb3Δ::crb3-WT-TAP isolate 2* | This study | Figure 3-Supp2 A |
| SPY9871 | *h- leu1-32 ade6-M216* *ura4Δ::10xtetO-ade6 trp1: nat-clr4p-NLS-TetR-2xflag-clr4ΔCD epe1Δ::kanMX6 crb3Δ::crb3-D198N-TAP isolate 1* | This study | Figure 3-Supp2 A |
| SPY9872 | *h- leu1-32 ade6-M216* *ura4Δ::10xtetO-ade6 trp1: nat-clr4p-NLS-TetR-2xflag-clr4ΔCD epe1Δ::kanMX6 crb3Δ::crb3-D198N-TAP isolate 2* | This study | Figure 3-Supp2 A |
| SPY9873 | *h+ leu1-32 ade6-216 ura4Δ::10xtetO-ura4-gfp chk1Δ::chk1-HA isolate 1* | This study | Figure 5G |
| SPY9874 | *h+ leu1-32 ade6-216 ura4+ chk1Δ::chk1-HA isolate 2* | This study | Figure 3E,F  Figure 3Supp2B,D,E  Figure 6Supp1 B-F |
| SPY9875 | *h+ leu1-32 ade6-216 ura4+ chk1Δ::chk1-HA dhp1-1 (:ura4) isolate 1* | This study | Figure 5G  Figure 6Supp1 B-F |
| SPY9876 | *h+ leu1-32 ade6-216 ura4+ chk1Δ::chk1-HA dhp1-1 (:ura4) isolate 2* | This study | Figure 6Supp1 B-F |
| SPY9877 | *h+ leu1-32 ade6-216 ura4Δ::10xtetO-ura4-gfp chk1Δ::chk1-HA grc3Δ::grc3-V70M isolate 1* | This study | Figure 5G  Figure 6Supp1 B-F |
| SPY9878 | *h+ leu1-32 ade6-216 ura4+ chk1Δ::chk1-HA grc3Δ::grc3-V70M isolate 2* | This study | Figure 5H,I  Figure 6Supp1 B-F |
| SPY5470 | *fWP44 h+ leu1-32 ade6-M216 clr4Δ::kanMX6* | Lab stock | Figure 3H  Figure 6Supp1A |
| SPY9879 | *h+ leu1-32 ade6-210 ura4Δ::10xtetO-ura4-gfp crb3Δ::crb3-WT-TAP isolate 1* | This study | Figure 3E,F  Figure 3Supp2B,D,E |
| SPY9880 | *h+ leu1-32 ade6-210 ura4Δ::10xtetO-ura4-gfp crb3Δ::crb3-WT-TAP isolate 2* | This study | Figure 3E,F  Figure 3Supp2B,D,E |
| SPY9881 | *h+ leu1-32 ade6-210 ura4Δ::10xtetO-ura4-gfp crb3Δ::crb3-WT-TAP grc3Δ::grc3-V70M isolate 1* | This study | Figure 3E,F  Figure 3Supp2B,D,E |
| SPY9882 | *h+ leu1-32 ade6-210 ura4Δ::10xtetO-ura4-gfp crb3Δ::crb3-WT-TAP grc3Δ::grc3-V70M isolate 2* | This study | Figure 3E,F  Figure 3Supp2B,D,E |
| SPY9883 | *h- leu1-32 ade6-M210 ura4-D18 his3-Dr crb3Δ::crb3-WT-TAP isolate 1* | This study | Figure 3Supp1 C,D,E |
| SPY9884 | *h- leu1-32 ade6-M210 ura4-D18 his3-Dr crb3Δ::crb3-WT-TAP isolate 2* | This study | Figure 3Supp1 C,D,E |
| SPY9885 | *h- leu1-32 ade6-M210 ura4-D18 his3-Dr crb3Δ::crb3-D198N-TAP isolate 1* | This study | Figure 3Supp1 C,D,E |
| SPY9886 | *h- leu1-32 ade6-M210 ura4-D18 his3-Dr crb3Δ::crb3-D198N-TAP isolate 2* | This study | Figure 3Supp1 C,D,E |
| SPY5465 | *fWP44 h+ leu1-32 ade6-M216* *ago1Δ::kanMX6* | Lab stock | Figure 2F,G |
| SPY3225 | *KGP425 h- leu1-32 ade6-M210 ura4-D18 his3-Dr clr4Δ::hphMX6 isolate 1* | Lab stock | Figure 2F,G |
| SPY3226 | *KGP425 h- leu1-32 ade6-M210 ura4-D18 his3-Dr clr4Δ::hphMX6 isolate 2* | Lab stock | Figure 2F,G |
| SPY9887 | *WP44 h+ leu1-32 ade6-M210/M216 crb3Δ::crb3-D198N isolate 1* | This study | Figure 2F,G |
| SPY9888 | *WP44 h+ leu1-32 ade6-M210/M216 crb3Δ::crb3-D198N isolate 2* | This study | Figure 2F,G |
| SPY9889 | *WP44 h+ leu1-32 ade6-M210/M216 crb3Δ::crb3-D198N ago1Δ::kanMX6 isolate 1* | This study | Figure 2F,G |
| SPY9890 | *WP44 h+ leu1-32 ade6-M210/M216 crb3Δ::crb3-D198N ago1Δ::kanMX6 isolate 2* | This study | Figure 2F,G |
| SPY9891 | *h- leu1-32 ade6-M216 ura4Δ::10xtetO-ade6 trp1: nat-clr4p-NLS-TetR-2xflag-clr4ΔCD epe1Δ::kanMX6 dhp1-1(:ura4) isolate 1* | This study | Figure 4A,B |
| SPY9892 | *h- leu1-32 ade6-M216 ura4Δ::10xtetO-ade6 trp1: nat-clr4p-NLS-TetR-2xflag-clr4ΔCD epe1Δ::kanMX6 dhp1-1(:ura4) isolate 3* | This study | Figure 4A,B |
| SPY9893 | *h- leu1-32 ade6-M216 ura4Δ::10xtetO-ade6 trp1: nat-clr4p-NLS-TetR-2xflag-clr4ΔCD epe1Δ::kanMX6 dhp1-1(:ura4) isolate 4* | This study | Figure 4A |
| SPY9894 | *h+ leu1-32 ade6-M210/M216 ura4Δ::10xtetO-ura4-GFP trp1: nat-clr4p-NLS-TetR-2xflag-clr4ΔCD epe1Δ::kanMX6 isolate 1* | This study | Figure 3H  Figure 5B,C,D,E  Figure 5Supp1B  Figure 6Supp1A |
| SPY9895 | *h+ leu1-32 ade6-M210/M216 ura4Δ::10xtetO-ura4-GFP trp1: nat-clr4p-NLS-TetR-2xflag-clr4ΔCD epe1Δ::kanMX6 isolate 2* | This study | Figure 3H  Figure 5B,C,D,E  Figure 5Supp1B  Figure 5Supp1 C  Figure 6Supp1A |
| SPY9896 | *h+ leu1-32 ade6-M210/M216 ura4Δ::10xtetO-ura4-GFP trp1: nat-clr4p-NLS-TetR-2xflag-clr4ΔCD epe1Δ::kanMX6 grc3Δ::grc3-V70M isolate 1* | This study | Figure 3H  Figure 5B,C,D,E  Figure 5Supp1B  Figure 6Supp1A |
| SPY9897 | *h+ leu1-32 ade6-M210/M216 ura4Δ::10xtetO-ura4-GFP trp1: nat-clr4p-NLS-TetR-2xflag-clr4ΔCD epe1Δ::kanMX6 grc3Δ::grc3-V70M isolate 2* | This study | Figure 3H  Figure 5B,C,D,E  Figure 5Supp1B  Figure 5Supp1 C  Figure 6Supp1A |
| SPY9898 | *h+ leu1-32 ade6-M210/M216 ura4Δ::10xtetO-ura4-GFP trp1: nat-clr4p-NLS-TetR-2xflag-clr4ΔCD epe1Δ::kanMX6 caf1Δ::hphMX6 isolate 1* | This study | Figure 5B,C,D,E |
| SPY9899 | *h+ leu1-32 ade6-M210/M216 ura4Δ::10xtetO-ura4-GFP trp1: nat-clr4p-NLS-TetR-2xflag-clr4ΔCD epe1Δ::kanMX6 caf1Δ::hphMX6 isolate 2* | This study | Figure 5B,C,D,E |
| SPY9900 | *h+ leu1-32 ade6-M210/M216 ura4Δ::10xtetO-ura4-GFP trp1: nat-clr4p-NLS-TetR-2xflag-clr4ΔCD epe1Δ::kanMX6 caf1Δ::hphMX6 grc3Δ::grc3-V70M isolate 1* | This study | Figure 5B,C,D,E |
| SPY9901 | *h+ leu1-32 ade6-M210/M216 ura4Δ::10xtetO-ura4-GFP trp1: nat-clr4p-NLS-TetR-2xflag-clr4ΔCD epe1Δ::kanMX6 caf1Δ::hphMX6 grc3Δ::grc3-V70M isolate 2* | This study | Figure 5B,C,D,E |
| SPY5099 | *h- leu1-32 ade6-M210 ura4Δ::10XtetO-ura4-GFP isolate 1* | Ref. 1 | Figure 3H  Figure 5B,C,D,E  Figure 5Supp1B  Figure 6Supp1A |
| SPY9905 | *h+ leu1-32 ade6-M210/M216 ura4Δ::10xtetO-ura4-GFP trp1: nat-clr4p-NLS-TetR-2xflag-clr4ΔCD epe1Δ::kanMX6 dhp1-1 (:ura4) isolate 2* | This study | Figure 5Supp1 C |
| SPY9907 | *h+ leu1-32 ade6-M210/M216 ura4Δ::10xtetO-ura4-GFP trp1: nat-clr4p-NLS-TetR-2xflag-clr4ΔCD epe1Δ::kanMX6 grc3Δ::grc3-V70M dhp1-1 (:ura4) isolate 2* | This study | Figure 5Supp1 C |
| SPY5362 | *h- leu1-32 ade6-M210/M216 ura4Δ::10xtetO-ade6 trp1: nat-clr4p-NLS-TetR-2xflag-clr4ΔCD epe1Δ::kanMX6 ago1Δ::hphMX6* *isolate 2* | Ref. 1 | Figure 5Supp1 C |
| SPY9909 | *h+ leu1-32 ade6-M210/M216 ura4Δ::10xtetO-ura4-GFP trp1: nat-clr4p-NLS-TetR-2xflag-clr4ΔCD epe1Δ::kanMX6* *swi6Δ::hphMX6 isolate 2* | This study | Figure 5Supp1 C |
| SPY9911 | *h+ leu1-32 ade6-M210/M216 ura4Δ::10xtetO-ura4-GFP trp1: nat-clr4p-NLS-TetR-2xflag-clr4ΔCD epe1Δ::kanMX6* *swi6Δ::hphMX6 grc3Δ::grc3-V70M isolate 2* | This study | Figure 5Supp1 C |
| SPY3 | *SPG1218 mat1Msmto leu1-32 his2 L(Bgl II)::ade6 ade6-DN/N* | Ref. 4 | Figure 5H,I  Figure 6A-H |
| SPY7305 | *SPG1218 mat1Msmto leu1-32 his2 L(Bgl II)::ade6 ade6-DN/N, clr4∆::kanMX6 isolate 1* | Lab stock | Figure 5H,I  Figure 6A-H |
| SPY9912 | *SPG1218 mat1Msmto leu1-32 his2 L(Bgl II)::ade6 ade6-DN/N* *grc3Δ::grc3-V70M isolate 1* | This study | Figure 5H,I  Figure 6A-H |
| SPY9913 | *SPG1218 mat1Msmto leu1-32 his2 L(Bgl II)::ade6 ade6-DN/N* *grc3Δ::grc3-V70M isolate 2* | This study | Figure 5H,I  Figure 6A-H |
| SPY9914 | *SPG1218 mat1Msmto leu1-32 his2 L(Bgl II)::ade6 ade6-DN/N dhp1-1 (:ura4) isolate 1* | This study | Figure 5H,I  Figure 6A-H |
| SPY9915 | *SPG1218 mat1Msmto leu1-32 his2 L(Bgl II)::ade6 ade6-DN/N dhp1-1 (:ura4) isolate 2* | This study | Figure 5H,I  Figure 6A-H |
| SPY9916 | *SPG1218 mat1Msmto leu1-32 his2 L(Bgl II)::ade6 ade6-DN/N clr4Δ::kanMX6-5'(1kb)-3xflag-clr4-W31G isolate 1* | This study | Figure 6A-H |
| SPY9917 | *SPG1218 mat1Msmto leu1-32 his2 L(Bgl II)::ade6 ade6-DN/N clr4Δ::kanMX6-5'(1kb)-3xflag-clr4-W31G isolate 2* | This study | Figure 6A-H |
| SPY9918 | *h- leu1-32 ade6-M216 ura4Δ::10xtetO-ade6 trp1: nat-clr4p-NLS-TetR-2xflag-clr4ΔCD epe1Δ::kanMX6 cid14Δ::hphMX6* | This study | Figure 5Supp1 A |
| SPY9919 | *h- leu1-32 ade6-M216 ura4Δ::10xtetO-ade6 trp1: nat-clr4p-NLS-TetR-2xflag-clr4ΔCD epe1Δ::kanMX6 cid14Δ::hphMX6 grc3Δ::grc3-V70M* | This study | Figure 5Supp1 A |
| SPY9920 | *h+ leu1-32 ade6-M210/M216 ura4Δ::10xtetO-ura4-GFP trp1: nat-clr4p-NLS-TetR-2xflag-clr4ΔCD epe1Δ::kanMX6 cid14Δ::hphMX6 isolate 1* | This study | Figure 5Supp1B |
| SPY9921 | *h+ leu1-32 ade6-M210/M216 ura4Δ::10xtetO-ura4-GFP trp1: nat-clr4p-NLS-TetR-2xflag-clr4ΔCD epe1Δ::kanMX6 cid14Δ::hphMX6 isolate 2* | This study | Figure 5Supp1B |
| SPY9922 | *h+ leu1-32 ade6-M210/M216 ura4Δ::10xtetO-ura4-GFP trp1: nat-clr4p-NLS-TetR-2xflag-clr4ΔCD epe1Δ::kanMX6 cid14Δ::hphMX6 grc3Δ::grc3-V70M isolate 1* | This study | Figure 5Supp1B |
| SPY9923 | *h+ leu1-32 ade6-M210/M216 ura4Δ::10xtetO-ura4-GFP trp1: nat-clr4p-NLS-TetR-2xflag-clr4ΔCD epe1Δ::kanMX6 cid14Δ::hphMX6 grc3Δ::grc3-V70M isolate 2* | This study | Figure 5Supp1B |
| SPY9926 | *h? leu1-32 ade6-M216 ura4Δ::10xtetO-ade6 trp1: nat-clr4p-NLS-TetR-2xflag-clr4ΔCD epe1Δ::kanMX6 dis3Δ::dis3-54 isolate 1* | This study | Figure 4Supp1 |
| SPY9927 | *h? leu1-32 ade6-M216 ura4Δ::10xtetO-ade6 trp1: nat-clr4p-NLS-TetR-2xflag-clr4ΔCD epe1Δ::kanMX6 dis3Δ::dis3-54 isolate 2* | This study | Figure 4Supp1 |
| SPY9928 | *h? leu1-32 ade6-M216 ura4Δ::10xtetO-ade6 trp1: nat-clr4p-NLS-TetR-2xflag-clr4ΔCD epe1Δ::kanMX6 dis3Δ::dis3-54 isolate 3* | This study | Figure 4Supp1 |
| SPY5081 | *h- leu1-32 ade6-M210 ura4Δ::10xtetO-ade6, clr4Δ::kanMX6 isolate 1* | Lab stock | Figure 2D,E  Figure 4B |
| SPY5082 | *h- leu1-32 ade6-M210 ura4Δ::10xtetO-ade6, clr4Δ::kanMX6 isolate 2* | Lab stock | Figure 2D,E  Figure 4B |
| SPY10093 | *h+ leu1-32 ade6-216 ura4? chk1Δ::chk1-HA grc3Δ::grc3-V70M dhp1-1 (:ura4) isolate 1* | This study | Figure 5G |
| SPY1574 | *h+ leu1-32 ade6-M216 ura4-D18 his3-Dr swi6Δ::natMX6* | Lab stock | Figure 5G |
| SPY10095 | *h+ leu1-32 ade6-216 ura4? chk1Δ::chk1-HA grc3Δ::grc3-V70M swi6Δ::natMX6 isolate 1* | This study | Figure 5G |
| SPY10097 | *h? leu1-32::ura4-pgrc3-grc3+-tgrc3 ade6-M216 ura4Δ::10xtetO-ade6 trp1: nat-clr4p-NLS-TetR-2xflag-clr4ΔCD epe1Δ::kanMX6 grc3Δ::grc3-G409E isolate 1* | This study | Figure 4C,D |
| SPY10099 | *h? leu1-32::ura4-pgrc3-grc3-K252A,S253A-tgrc3 ade6-M216 ura4Δ::10xtetO-ade6 trp1: nat-clr4p-NLS-TetR-2xflag-clr4ΔCD epe1Δ::kanMX6 grc3Δ::grc3-G409E isolate 1* | This study | Figure 4C,D |

References

1. K. Ragunathan, G. Jih, D. Moazed, Epigenetic inheritance uncoupled from sequence-specific recruitment. *Science* **348**, 1258699 (2015).

2. G. Thon, P. Bjerling, C. M. Bunner, J. Verhein-Hansen, Expression-state boundaries in the mating-type region of fission yeast. *Genetics* **161**, 611-622 (2002).

3. P. Bjerling *et al.*, Functional divergence between histone deacetylases in fission yeast by distinct cellular localization and in vivo specificity. *Mol Cell Biol* **22**, 2170-2181 (2002).

4. J. Nakayama, A. J. Klar, S. I. Grewal, A chromodomain protein, Swi6, performs imprinting functions in fission yeast during mitosis and meiosis. *Cell* **101**, 307-317. (2000).
