# Supplementary material for: A conserved RNA degradation complex required for spreading and epigenetic inheritance of heterochromatin
Source: eLife. 2020 Jun 3;9:e54341. doi: 10.7554/eLife.54341 (PMC7269676; doi:10.7554/eLife.54341)
Supplement: Supplementary file 2. — List of DNA oligonucleotides used in RT-qPCR, ChIP-qPCR and northern blots. [file elife-54341-supp2.docx]

Table S2. List of oligonucleotides used in this study.

| **Oligo** | **Sequence** | **Description** |
| --- | --- | --- |
| AS131 | AAGGAATGTGCCTCGTCAAATT | *dg* FWD, qPCR, sRNA northern |
| AS132 | TGCTTCACGGTATTTTTTGAAATC | *dg* REV, qPCR, RT, sRNA northern |
| GJ173 | ATTGACGCCGGTGTTAGTGTAGGT | *fbp1* FWD, qPCR |
| GJ174 | TGACACGATGACCTGTGGTAAGCA | *fbp1* REV, qPCR |
| GSqPCR_27 | AGACGGTTCAAACTCCCTCT | *tam14* FWD, qPCR |
| GSqPCR_28 | ACGAGGAATCTTGGTAGGAGGA | *tam14* REV, qPCR |
| AS133 | GTATTTGGATTCCATCGGTACTATGG | *dh* FWD, qPCR |
| AS134 | ACTACATCGACACAGAAAAGAAAACAA | *dh* REV, qPCR |
| XW411 | CATATCAATTACAAACATATTGTTTGCTGC | *cenH-mat2*, qPCR |
| XW412 | GCTTCATGTTAAGTTGATTGGTGTAATTAG | *cenH-mat2*, qPCR |
| GSqPCR_67 | ACAGGGCGTCAACCATGACA | *tlh1* FWD, qPCR |
| GSqPCR_68 | TTGACGGCTTGCACACGACT | *tlh1* REV, qPCR |
| NI1234 | GAGGTAAAGCGAATGATTAGAGGT | 28S rRNA, qPCR |
| NI1235 | TTAAAGTTTGAGAATAGGTTGAGGAAA | 28S rRNA, qPCR |
| NI1226/1227 | GTTGTTGCAGTTAAAAAGCTCGTA | 18S rRNA, qPCR |
| NI1226/1227 | ATGACCAGTAAACACGCCTTG | 18S rRNA, qPCR |
| GSqPCR_71 | CGTCCATACCGTGAGTGATACC | *gfp* REV, qPCR, RT |
| GSqPCR_72 | CGGTCCAGTTTTGTTGCCAGAC | *gfp* FWD, qPCR |
| GSqPCR_87 | ACTGAACGTACTCCGAGACC | *mat3* FWD (position 6), qPCR |
| GSqPCR_88 | TCGCATACGTACTTCCTTTGA | *mat3* REV (position 6), qPCR |
| AS49 | CAACCCTCAGCTTTGGGTCTTG | *act1* FWD, qPCR |
| AS50 | TCCTTTTGCATACGATCGGCAATAC | *act1* REV, qPCR, RT |
| GSqPCR_129 | CAAATCACCGCTTTTGAGATTAATCGT | *IR-L:ade6* (position 1) FWD, qPCR |
| GSqPCR_130 | CAACAGGCGGCCAAGCAATTT | *IR-L:ade6* (position 1) REV, qPCR |
| GSqPCR_137 | GCAGTTTAGACGGAAAAGTTTATGC | *ade6* FWD (position 2), qPCR |
| GSqPCR_138 | ATTGAGAAGGGAAGACGAGCAGG | *ade6* REV (position 2), qPCR, qRT |
| GSqPCR_125 | AGGACCCATACAACAACGGA | *RE-II* (position 3) FWD, qPCR |
| GSqPCR_126 | TGCCAAGCAAGCAACTGTTTA | *RE-II* (position 3) REV, qPCR |
| GSqPCR_97 | TGAGCATTTGGCTGAAGCACC | *mat2* FWD (position 4), qPCR |
| GSqPCR_98 | CCAAATACACCAAGCCATAATACGCAC | *mat2* REV (position 4), qPCR |
| GSqPCR_139 | GATGATAACGGATCTAGCTTCGCCA | *cenH* FWD (position 5), qPCR |
| GSqPCR_140 | GCCTCTCTCTATATATCAGATATAAAGATGCG | *cenH* REV (position 5), qPCR, qRT |
| GSqPCR_89 | GTGCTTCAGCCAAATGCTCA | *mat2* FWD, qPCR |
| GSqPCR_90 | ACTGAACCCTGCTTATATGTAGTT | *mat2* REV, qPCR, RT |
