# Supplementary material for: A conserved RNA degradation complex required for spreading and epigenetic inheritance of heterochromatin
Source: eLife. 2020 Jun 3;9:e54341. doi: 10.7554/eLife.54341 (PMC7269676; doi:10.7554/eLife.54341)
Supplement: Transparent reporting form [file elife-54341-transrepform.pdf]

1st Floor
24 Hills Road
Cambridge CB2 1JP, UK

P 01223 855340
W elifesciences.org
T @elife

eLife's transparent reporting form

We encourage authors to provide detailed information *within their submission* to facilitate the interpretation and replication of experiments. Authors can upload supporting documentation to indicate the use of appropriate reporting guidelines for health-related research (see EQUATOR Network), life science research (see the BioSharing Information Resource), or the ARRIVE guidelines for reporting work involving animal research. Where applicable, authors should refer to any relevant reporting standards documents in this form.

If you have any questions, please consult our Journal Policies and/or contact us: editorial@elifesciences.org.

Sample-size estimation

- You should state whether an appropriate sample size was computed when the study was being designed
- You should state the statistical method of sample size computation and any required assumptions
- If no explicit power analysis was used, you should describe how you decided what sample (replicate) size (number) to use

Please outline where this information can be found within the submission (e.g., sections or figure legends), or explain why this information doesn't apply to your submission:

1) Screening was performed on 80,000 colonies and 83 mutants of the desired phenotype were picked for further analysis. Pooled linkage analysis (PLA) was performed to identify variants co-segregating with the phenotype of interest. The number of spores in PLA was estimated based on the expected frequency of mutations generated by 3% EMS treatment, so as to exclude all background variants, which do not co-segregate with the desired trait. A minimum depth of sequencing of 3 million reads per mutant pool was set as a threshold for analysis. The wild-type parental strains were sequenced at an increased depth of 10 million reads per genome. Variants were identified by the pipeline Mudi. Hits were selected as variants present in at least 90% of the reads at a genomic region and further prioritized by presence in coding regions, excluding silent mutations.
2) For qRT and ChIP-qPCR, three biological replicates were used for quantification.
3) For ChIP-seq, two independently derived clones were used to control for clonal variability.
4) Every TMT-MS experiment was performed once and z-scores were calculated for proteins in each run.

Replicates

- You should report how often each experiment was performed
- You should include a definition of biological versus technical replication
- The data obtained should be provided and sufficient information should be provided to indicate the number of independent biological and/or technical replicates
- If you encountered any outliers, you should describe how these were handled
- Criteria for exclusion/inclusion of data should be clearly stated

- High-throughput sequence data should be uploaded before submission, with a private link for reviewers provided (these are available from both GEO and ArrayExpress)

Please outline where this information can be found within the submission (e.g., sections or figure legends), or explain why this information doesn't apply to your submission:

1) Western and Northern blotting were performed in at least two biological replicates. Effort was made, where possible, to use two independently derived clones.
2) In qRT and ChIP-qPCR experiments, two technical replicates were routinely used to control for qPCR technical errors.
3) In TMT-MS data analysis, proteins detected with a single peptide were excluded from quantification (see Methods section for further analysis).
4) GEO link for high-throughput sequencing data:
https://www.ncbi.nlm.nih.gov/geo/query/acc.cgi?acc=GSE140920
Token for reviewers: ytifkwkadtuvvur
5) Link to raw data files on Mendeley:
https://data.mendeley.com/datasets/bgd3sjp9dt/draft?a=fb4c0e75-5de1-419e-bdf0-75f4f3731745

Statistical reporting

- Statistical analysis methods should be described and justified
- Raw data should be presented in figures whenever informative to do so (typically when N per group is less than 10)
- For each experiment, you should identify the statistical tests used, exact values of N, definitions of center, methods of multiple test correction, and dispersion and precision measures (e.g., mean, median, SD, SEM, confidence intervals; and, for the major substantive results, a measure of effect size (e.g., Pearson's r, Cohen's d)
- Report exact p-values wherever possible alongside the summary statistics and 95% confidence intervals. These should be reported for all key questions and not only when the p-value is less than 0.05.

Please outline where this information can be found within the submission (e.g., sections or figure legends), or explain why this information doesn't apply to your submission:

1) For each qRT and ChIP-qPCR experiment, a bar-graph of the mean of three biological replicates is displayed and error bars indicate SD (also stated in the figure legends).
2) For TMT-MS plots, a z-score for candidate protein is shown (calculation of z-scores is described in the Methods).
3) For ChIP-seq data, all reads are normalized to the total number of reads in each sample and displayed as reads per million (RPM).

(For large datasets, or papers with a very large number of statistical tests, you may upload a single table file with tests, Ns, etc., with reference to sections in the manuscript.)

Group allocation

- Indicate how samples were allocated into experimental groups (in the case of clinical studies, please specify allocation to treatment method); if randomization was used, please also state if restricted randomization was applied
- Indicate if masking was used during group allocation, data collection and/or data analysis

Please outline where this information can be found within the submission (e.g., sections or figure legends), or explain why this information doesn't apply to your submission:

n/a

Additional data files ("source data")

- We encourage you to upload relevant additional data files, such as numerical data that are represented as a graph in a figure, or as a summary table
- Where provided, these should be in the most useful format, and they can be uploaded as "Source data" files linked to a main figure or table
- Include model definition files including the full list of parameters used
- Include code used for data analysis (e.g., R, MatLab)
- Avoid stating that data files are "available upon request"

Please indicate the figures or tables for which source data files have been provided:

1) Screen data were analyzed by the published pipeline Mudi (see Methods).
2) Raw data for TMT-MS plots in Figure 3B and Figure S4D are provided as Tables S3 and S4 (excel files).

eLife Sciences Publications, Ltd is a limited liability non-profit non-stock corporation incorporated in the State of Delaware, USA, with company number 5030732, and is registered in the UK with company number FC030576 and branch number BR015634 at the address 1st Floor, 24 Hills Road, Cambridge CB2 1JP | August 2014
